# Mental health and satisfaction with partners: a longitudinal analysis in the UK

**DOI:** 10.1186/s40359-022-00723-w

**Published:** 2022-01-27

**Authors:** Paul Downward, Simona Rasciute, Harish Kumar

**Affiliations:** 1 School of Sport, Exercise and Health Sciences, Loughborough University, Ashby Road, Loughborough, LE11 3TU UK; 2 School of Business and Economics, Loughborough University, Ashby Road, Loughborough, LE11 3TU UK; 3 Department of Economics, Policy and International Business, Manchester Metropolitan University, Manchester, M15 6BH UK

**Keywords:** Mental health, Personal relationships, Causality, Panel data

## Abstract

**Background:**

Current UK health policy stresses treating health as an asset to underpin and promote a more inclusive and productive society. The quality of personal relationships is essential for overall quality of life. The social determinants of health (SDH) literature shows that poor mental health and well-being are linked to weaker personal and social connections for individuals, families, and society. The causal impact that mental health has on satisfaction with partners is less understood but requires investigation.

**Methods:**

The causal relationship between mental health and satisfaction with partners is examined drawing on the United Kingdom’s British Household Panel Survey from 1991 to 2008. A total sample of 9,024 individuals in dyadic couples comprising 42,464 observations was analysed using fixed-effects and instrumental variable fixed-effects panel data estimation.

**Results:**

Lower mental health is associated with a lower satisfaction with partners. However, some causal evidence of lower mental health reducing satisfaction with partners is present for males.

**Discussion:**

For females, relationship satisfaction is more likely to influence mental health. For males there is a potential ‘vicious circle’ between satisfaction with partners and mental health.

**Conclusions:**

Investment in mental health provision can improve satisfaction with partners which in turn will further enhance health and well-being.

## Background

This paper investigates the relationship between mental health and satisfaction with partners in the United Kingdom (UK). It finds that lower mental health is associated with a lower satisfaction with partners generally but there is some causal evidence of lower mental health reducing satisfaction with partners for males. The context of the investigation is that the World Health Organisation (WHO) Rio Political Declaration in 2011 called for a global political commitment to address the Social Determinants of Health (SDH) and for national health policies and strategies to reduce the health inequalities deriving from the conditions in which people are born, grow, live, work and age [1]. This perspective is now acknowledged in the United Kingdom (UK), where it has recently been argued that health policy needs to redirect emphasis from treating diseases when they arise, to focussing on actions that maintain health and flourishing over the life course [2, 3], treating health as an asset [4]. Importantly, it has long been suggested that the family’s health and well-being can be a barometer for measuring the nation’s health and well-being [5] and UK social welfare policy now recognises the importance of raising well-being, through the quality of personal relationships [6–8]. It follows that in adopting an asset-based approach to public health [2], better understanding the links between satisfaction with partners and their health is important.

There are longstanding theoretical reasons to expect a link between relationships generally, well-being and health [9–12]. Relationships can offset loneliness [13] and provide a basis for enjoyable shared social interactions and emotional pleasure [14], which is more likely to be the case with attachment relationships, for example, between household members who are close and extremely important to one another [15, 16]. Satisfaction with partners, traditionally in marriage, is identified as being important for adults [17]. The marital resource model indicates that marriage, but by implication partnerships, provides social, psychological and economic resources that contribute to better health which would not be available to those living on their own [18].

Two strands of empirical work addressing these issues can be identified. One strand focuses on the characteristic of being in a partnership as an implied determinant of health and wellbeing. For example, being married or cohabiting is associated with improved health, lower morbidity and mortality [19, 20], less functional limitations [21] and lower mortality for causes of deaths that have a strong behavioural basis, such as lung cancer and cirrhosis [18]. Married individual are less likely to smoke [19], more likely to eat healthier diets, less likely to drink heavily and to take risks that increase the probability of accidents and injuries [18]. These effects are also found to vary between genders [22–24] as well as age, with the health gap between married and unmarried individuals being the largest at the age of 55–59 years [25].

Another strand of literature focusses directly on satisfaction with the dyadic relationship and its association with health and well-being, exploring potential differences between males and females. It has been found that depression at a later point in time, for both males and females in a couple, can stem from previously experienced marital dissatisfaction [26], that higher overall life satisfaction for both male and female partners is associated with higher marital quality [27], and that marital strain can accelerate the decline in self-rated health for both ageing males and female partners [28]. However, research has also indicated that wives’ well-being is more closely associated with marital satisfaction than their partners [29].

Significantly, less research has focussed on the implied role of health and well-being as a determinant of satisfaction with partners. Nonetheless, it has been shown that higher levels of depression can predict lower marital satisfaction [52] and this might be more relevant for males [30, 31]. Moreover, the reverse influence of marital satisfaction on depression has been shown to be more relevant for females [31]. More generally, reviews of studies show that greater satisfaction with partners is related to better health [32].

Finally, other contextual factors have been shown to be important factors that shape the role of family relationships on well-being and health. These include income [33], educational level and occupational hierarchy [34–37].

Drawing on the above policy context and literature this study seeks to contribute to better understanding the relationship between mental health and the satisfaction with partners for individuals who are married or in a civil partnership based on nationally representative longitudinal data. More specifically, the paper seeks to make a number of contributions. First, it responds to the call for more longitudinal studies that provide stronger causal inferences on such relationships [32]. The focus is upon mental health because of its central importance for relationship building and well-being [38]. Second, analysis is undertaken for both males and females because of the recognised differences between the genders [22–24] and, as noted above, the research identifies potentially different associations for males and females between depression and satisfaction with partners [30, 31]. Moreover, there are a variety of different reasons to expect gender differences. Females might be more comfortable discussing their emotional distress [22]. Females, compared to males, moreover tend to manage more roles within and outside the family and, consequently, in the presence of illness, will experience more role disruption [22]. Finally, the dyadic roles of partners is also accounted for in the analysis because although some research does not identify an association between cross-partner satisfaction and own outcomes [27], cross-partner satisfaction effects have been identified in other research [30].

## Methods

A longitudinal observational research design is employed based upon data from the British Household Panel Survey (BHPS). The BHPS is a longitudinal social survey of households and individuals living in the UK. It began in 1991 and lasted until 2008, when it was superseded by the Understanding Society Survey (USS). The first set of questions were implemented between late 1991 and early 1992 in Wave 1 and contained approximately 5,500 households with 10,300 individuals from England. In 1999, the survey was rolled out in Scotland and Wales, and in 2001 a sample from Northern Ireland was added. By Wave 11, the panel had grown to 10,500 households. The data cover a wide range of information including household and individual characteristics, socio-demographics, health and education, finances, and social activities. The BHPS is employed in this research as it explicitly investigates satisfaction with partners following the same representative sample of individuals for 10 years between 1998 and 2008. The analysis focusses on a dyadic sample of 9,024 individuals who are either married or in a civil partnership in each wave of the survey, producing a total sample of 42,464 observations.

The mental health measure for this study is the 12 item General Health Questionnaire (GHQ12) [39]. The Likert version is used for the analysis [40, 41]. The GHQ12 measure is the most commonly used screening instrument for the detection of common mental disorders as well as being a more general measure of psychological wellbeing [42, 43]. The GHQ12 is a self-reported measure of mental health that comprises 12 questions regarding the respondent's emotional and psychological health over the past few weeks that precede the interview [44]. Higher values of GHQ12 indicate reduced mental health. It should be recognised, however, that as a composite measure, GHQ12 score can potentially mask the influence of more nuanced and targeted insights that might arise from focussing on specific items within the scale [43].

Satisfaction with partners is measured in the BHPS through a question that asks individuals to rate explicitly how dissatisfied or satisfied they are with their spouse or partner. The responses range between 1–7, where 1 represents not at all satisfied and 7 represents completely satisfied. This variable measures one aspect of life-satisfaction, as a component of general well-being [45], and is linked to marital quality through being an expression of self-reported satisfaction with a relationship [32]. The measure is distinct from hedonic, experiential measures of well-being. These capture happiness or pleasure, such as the degree of happiness in a relationship as, for example, measured in the USS [46]. Satisfaction with partners is also distinct from the eudaimonic notion of well-being which is connected with the personal growth, development and functioning of individuals through their activities [47]. This latter aspect of well-being resonates most closely with the dimension of dyadic cohesion in which couples collectively engage in activities, and exchange of ideas through discussion [48]. It forms part of a broader model of marital adjustment in which dyadic consensus, dyadic satisfaction and affectional expression are also part [49]. Dyadic consensus identifies the degree to which a couple agrees on matters of importance to their relationship, dyadic satisfaction identifies the degree to which a couple is satisfied with their relationship, and affective expression shows the degree of demonstration of affection [50]. Within the context of this model, the current research focusses on the dyadic satisfaction dimension.

Confounding variables measuring the individual’s age [51], level of education, employment status [34–36], and income [33] are included in the analysis as they are identified to be important in the literature above. A measure of the presence of long-term illness is added because of the potential for pre-existing conditions to shape how changes in health influence relationship satisfaction [37].

Finally, because in a dyadic context there is the potential for an individuals’ partner satisfaction with them to influence their satisfaction with their partner, alternative specifications of the analysis were undertaken with the partners satisfaction with their partner included and excluded [30, 52]. All variables and descriptive statistics are presented in Table 1.

**Table 1:** Variable descriptions and characteristics of data

Variable	Description	Mean	Std. Dev	Min	Q1	Median	Q3	Max
Satisfaction	Satisfaction with partner (1—not at all to 7 completely)	6.401	1.005	1	6	7	7	7
Partner	Partner satisfaction	6.401	1.005	1	6	7	7	7
GHQ12	GHQ12 Likert scale (0 to 36)	10.877	5.061	0	7	10	13	36
GHQ12Partner	Partner GHQ12 Likert scale (0 to 36)	10.877	5.061	0	7	10	13	36
Sex	Sex (1 – male, 0 -female)	0.500	0.500	0	–	–	–	1
Age	Age in years	50.121	15.708	18	36	48	63	94
Higher Education	Has a degree or equivalent (1—yes, 0—no)	0.261	0.439	0	–	–	–	1
Alevel	Has A levels (1—yes, 0—no)	0.205	0.404	0	–	–	–	1
GCSE	Has GCSEs or other qualification(1—yes, 0—no)	0.336	0.472	0	–	–	–	1
Child 0–2	Number of children aged 0 to 2 years	0.100	0.316	0	0	0	0	3
Child 3–4	Number of children aged 3 to 4 years	0.107	0.325	0	0	0	0	3
Child 5–11	Number of children aged 5 to 11 years	0.382	0.720	0	0	0	1	4
Child 12–15	Number of children aged 12 to 15 years	0.148	0.431	0	0	0	0	3
Real Family Income	Real gross monthly household income (£000)	1.773	1.650	0.00	0.66	1.39	2.39	21.04
Self employed	Self-employed (1—yes, 0—no)	0.083	0.276	0	–	–	–	1
Employed	Employed full time (1—yes, 0—no)	0.521	0.500	0	–	–	–	1
Unemployed	Unemployed (1—yes, 0—no)	0.013	0.114	0	–	–	–	1
Retired	Retired (1—yes, 0—no)	0.264	0.441	0	–	–	–	1
Maternity leave	On maternity leave (1—yes, 0—no)	0.007	0.086	0	–	–	–	1
Family care	Caring for the family (1—yes, 0—no)	0.071	0.257	0	–	–	–	1
Full-time study	In full-time study (1—yes, 0—no)	0.004	0.061	0	–	–	–	1
Long-term sick	On long-term sick leave (1—yes, 0—no)	0.032	0.177	0	–	–	–	1
England	Respondent is from England (1—yes, 0—no)	0.583	0.493	0	–	–	–	1
Scotland	Respondent is from Scotland (1—yes, 0—no)	0.171	0.377	0	–	–	–	1
Wales	Respondent is from Wales (1—yes, 0—no)	0.153	0.360	0	–	–	–	1
n		42,464						

The data presented there for dyadic couples is very similar to that for the sample of 65,768 individuals who are either married or in a civil partnership but are not matched across waves of the survey. For example, the mean values for the dyadic and non-dyadic individuals respectively are 6.401 and 6.354 for Satisfaction, 10.877 and 11.071 for GHQ12, 50.121 and 50.124 for Age and 1.773 and 1.749 for Real Family Income. A further consequence of focussing on such dyadic partners in the sample is that analysis distinguishing between the variety of forms of relationship status on satisfaction with partners is not undertaken. The focus is on married couples or those in a civil partnership rather than individuals who are cohabiting, separated, divorced, widowed or never married.

To work towards a causal understanding of the effects of mental health on satisfaction with partners, fixed effects, and instrumental variable fixed-effects panel data estimators are employed. The fixed effects control for endogeneity associated with unobserved time invariant personal characteristics, while the instrumental variable fixed-effects panel data estimator is employed to additionally control for reverse causality.

To apply the instrumental variable fixed-effects estimator, instrumental variables need to be identified that are correlated with GHQ12 but primarily affect satisfaction with partners indirectly through the mental health variable. Furthermore, the instrumental variables cannot be correlated with the unobserved factors measured by the fixed effects [53]. In this study, first of all dummy variables for the countries in the UK (England, Scotland and Wales compared to Northern Ireland and the Channel Isles) in which the respondent resides are employed as instrumental variables. The country dummy variables, it is assumed, capture elements of the national variation in supply of health [54]. The devolution of governments in the countries of Scotland, Wales, and Northern Ireland in 1999, has led to different system of governance across the countries. The availability of panel data enables the use of the lagged value of mental health as an instrument. It is likely that past and current mental health will be strongly correlated. However, the use of the lagged value of mental health as an instrument depends on the lagged values themselves not belonging to the estimating equation and that they are sufficiently correlated with the simultaneously determined explanatory variable [55].

The adequacy of the instrumental variables is, thus, tested statistically. Their relevance was examined through F-tests assessing their joint significance in a regression of the measure of mental health on the instrumental variables and other confounding variables. The potential significance of the lag of mental health being directly included in the equation explaining satisfaction with the partner was assessed through t-tests. Finally, the validity of the instrumental variables was tested by the Sargan-Hansen test, which examines the independence of the instrumental variables from the errors of the instrumental variable regression [53]. Finally, robust variance–covariance matrix estimates were used to draw all inferences to control for heteroscedasticity in the cross-sectional component of the data [56]. Relevant ethical clearance was obtained from the authors’ institution and all methods were applied in accordance with the relevant guidelines and regulations.

## Results

Tables 2 and 3 presents regression results for the fixed effects panel data estimator and the instrumental variable fixed effects panel data estimator, in which lagged mental health and country dummy variables are used as instruments, respectively. The estimates focus on the males and females in the couple and include and exclude measures of each other’s satisfaction as well as each other’s GHQ12 scores as confounders. The tests for the relevance and validity of the instruments are reported at the bottom of the tables. Collectively, the insignificant t-tests of lagged GHQ12 in the equations for satisfaction at the bottom of Table 2 and the statistically significant F-statistics of the instruments in equations for GHQ12 and the insignificant Sargan-Hansen statistics in Table 3 indicate evidence in favour of the relevance and validity of the instruments used.

**Table 2:** Non-IV regression results of mental health on personal relationships

	Male	Female	Male with partner satisfaction	Female with partner satisfaction	Male with partner GHQ12	Female with partner GHQ12
GHQ12	− 0.0210***	− 0.0213***	− 0.0189***	− 0.0195***	− 0.0197***	− 0.0201***
	(− 8.70)	(− 11.77)	(− 8.20)	(− 11.12)	(− 8.15)	(− 11.11)
Partner	–	–	0.158***	0.185***	–	–
			(12.07)	(11.60)		
GHQ12Partner	–	–	–	–	− 0.00753***	− 0.00921***
					(− 5.24)	(− 4.70)
Age	− 0.0124***	− 0.0227***	− 0.00898***	− 0.0202***	− 0.0120***	− 0.0221***
	(− 5.30)	(− 8.54)	(− 3.96)	(− 7.81)	(− 5.14)	(− 8.32)
Higher education	− 0.165	− 0.156	− 0.122	− 0.162	− 0.171	− 0.168
	(− 0.90)	(− 1.05)	(− 0.71)	(− 1.10)	(− 0.92)	(− 1.14)
Alevel	− 0.0399	− 0.00761	− 0.0269	− 0.00242	− 0.0419	− 0.0124
	(− 0.27)	(− 0.05)	(− 0.19)	(− 0.02)	(− 0.28)	(− 0.09)
GCSE	− 0.0351	0.0771	− 0.0344	0.0707	− 0.0304	0.0753
	(− 0.48)	(0.87)	(− 0.48)	(0.84)	(− 0.41)	(0.87)
Child 0–2	− 0.0404*	− 0.0292	− 0.0369*	− 0.0180	− 0.0370*	− 0.0277
	(− 1.85)	(− 1.15)	(− 1.74)	(− 0.73)	(− 1.70)	(− 1.09)
Child 3–4	− 0.0825***	− 0.107***	− 0.0653***	− 0.0908***	− 0.0816***	− 0.106***
	(− 3.66)	(− 4.36)	(− 2.96)	(− 3.77)	(− 3.62)	(− 4.36)
Child 5–11	− 0.0647***	− 0.0942***	− 0.0505***	− 0.0822***	− 0.0662***	− 0.0936***
	(− 3.25)	(− 4.53)	(− 2.66)	(− 4.08)	(− 3.34)	(− 4.50)
Child 12–15	− 0.0984***	− 0.119***	− 0.0784***	− 0.101***	− 0.0978***	− 0.118***
	(− 3.52)	(− 3.99)	(− 2.91)	(− 3.51)	(− 3.51)	(− 3.97)
Real family income	0.00251	0.0178**	0.00275	0.0199**	0.00245	0.0167*
	(0.55)	(1.99)	(0.61)	(2.30)	(0.53)	(1.87)
Self employed	− 0.107	− 0.111	− 0.106	− 0.130	− 0.108	− 0.108
	(− 0.90)	(− 0.84)	(− 0.89)	(− 1.02)	(− 0.92)	(− 0.82)
Employed	− 0.0982	− 0.0117	− 0.106	− 0.0319	− 0.0995	− 0.0101
	(− 0.83)	(− 0.09)	(− 0.90)	(− 0.27)	(− 0.85)	(− 0.08)
Unemployed	− 0.0488	0.0428	− 0.0683	0.0164	− 0.0480	0.0426
	(− 0.37)	(0.31)	(− 0.52)	(0.12)	(− 0.37)	(0.31)
Retired	− 0.130	0.0328	− 0.142	0.0191	− 0.134	0.0304
	(− 1.10)	(0.26)	(− 1.19)	(0.16)	(− 1.14)	(0.24)
Family care	0.0397	− 0.00151	0.0522	− 0.0240	0.0437	− 0.00190
	(0.20)	(− 0.01)	(0.26)	(− 0.20)	(0.22)	(− 0.02)
Full-time study	− 0.239	− 0.0818	− 0.246	− 0.111	− 0.245	− 0.0847
	(− 1.52)	(− 0.56)	(− 1.58)	(− 0.79)	(− 1.55)	(− 0.58)
Long-term sick	− 0.0665	− 0.0213	− 0.0953	− 0.0373	− 0.0697	− 0.0225
	(− 0.49)	(− 0.15)	(− 0.70)	(− 0.28)	(− 0.52)	(− 0.16)
Maternity leave	–	0.101	–	0.0497	–	0.101
		(0.76)		(0.39)		(0.76)
Constant	7.524***	7.764***	6.307***	6.435***	7.580***	7.823***
	(41.64)	(41.20)	(31.60)	(31.06)	(42.03)	(41.52)
*n*	21,232	21,232	21,232	21,232	21,232	21,232
t-stat of lag GHQ12	− 1.54	− 0.96	− 1.4	− 0.89	− 1.44	− 0.86

Robust cluster t statistics in parentheses

^*^p < 0.1; **p < 0.05; ***p < 0.01

**Table 3:** IV regression results of mental health on personal relationships

	Male	Female	Male with partner satisfaction	Female with partner satisfaction	Male with partner GHQ12	Female with partner GHQ12
GHQ12	− 0.0774*	− 0.0274	− 0.0645^a^	− 0.0269	− 0.0740*	− 0.00545
	(− 1.84)	(− 0.30)	(− 1.59)	(− 0.29)	(− 1.77)	(− 0.07)
Partner	–	–	0.144***	0.183***	–	–
			(8.30)	(4.96)		
GHQ12Partner	–	–	–	–	− 0.000863	− 0.0117
					(− 0.16)	(− 0.87)
Age	− 0.00858**	− 0.0223***	− 0.00619*	− 0.0198***	− 0.00876***	− 0.0228***
	(− 2.35)	(− 3.61)	(− 1.85)	(− 3.40)	(− 2.58)	(− 4.85)
Higher education	− 0.130	− 0.148	− 0.0971	− 0.153	− 0.132	− 0.190
	(− 0.72)	(− 0.79)	(− 0.57)	(− 0.82)	(− 0.73)	(− 1.04)
Alevel	− 0.00766	− 0.00307	− 0.00200	0.00300	− 0.00975	− 0.0243
	(− 0.05)	(− 0.02)	(− 0.01)	(0.02)	(− 0.06)	(− 0.16)
GCSE	0.00959	0.0792	0.00156	0.0734	0.00755	0.0699
	(0.11)	(0.84)	(0.02)	(0.80)	(0.09)	(0.79)
Child 0–2	− 0.0292	− 0.0267	− 0.0282	− 0.0152	− 0.0295	− 0.0331
	(− 1.19)	(− 0.58)	(− 1.21)	(− 0.34)	(− 1.24)	(− 0.83)
Child 3–4	− 0.0797***	− 0.106***	− 0.0645***	− 0.0904***	− 0.0798***	− 0.107***
	(− 3.38)	(− 4.06)	(− 2.85)	(− 3.64)	(− 3.41)	(− 4.19)
Child 5–11	− 0.0637***	− 0.0957***	− 0.0510***	− 0.0842***	− 0.0640***	− 0.0900***
	(− 3.14)	(− 3.27)	(− 2.64)	(− 2.77)	(− 3.16)	(− 3.29)
Child 12–15	− 0.0959***	− 0.119***	− 0.0781***	− 0.101***	− 0.0959***	− 0.118***
	(− 3.34)	(− 3.98)	(− 2.84)	(− 3.52)	(− 3.35)	(− 3.95)
Real family income	0.00312	0.0185	0.00322	0.0207	0.00308	0.0148
	(0.66)	(1.36)	(0.70)	(1.58)	(0.65)	(1.09)
Self employed	− 0.145	− 0.104	− 0.137	− 0.122	− 0.143	− 0.123
	(− 1.23)	(− 0.63)	(− 1.17)	(− 0.75)	(− 1.22)	(− 0.79)
Employed	− 0.143	− 0.00695	− 0.142	− 0.0258	− 0.141	− 0.0209
	(− 1.22)	(− 0.05)	(− 1.20)	(− 0.18)	(− 1.20)	(− 0.15)
Unemployed	0.0414	0.0515	0.00600	0.0272	0.0363	0.0222
	(0.28)	(0.27)	(0.04)	(0.14)	(0.24)	(0.13)
Retired	− 0.187	0.0380	− 0.187	0.0257	− 0.184	0.0174
	(− 1.54)	(0.26)	(− 1.55)	(0.18)	(− 1.54)	(0.12)
Family care	0.0206	0.00750	0.0357	− 0.0128	0.0222	− 0.0232
	(0.11)	(0.04)	(0.18)	(− 0.07)	(0.11)	(− 0.14)
Full-time study	− 0.299*	− 0.0782	− 0.294*	− 0.106	− 0.296*	− 0.0940
	(− 1.81)	(− 0.50)	(− 1.84)	(− 0.70)	(− 1.81)	(− 0.60)
Long-term sick	0.0402	− 0.00131	− 0.00689	− 0.0129	0.0337	− 0.0698
	(0.25)	(− 0.00)	(− 0.04)	(− 0.04)	(0.21)	(− 0.24)
Maternity leave	–	0.106	–	0.0571	–	0.0884
		(0.68)		(0.36)		(0.59)
Constant	7.913***	7.806***	6.726***	6.505***	7.897***	7.739***
	(23.02)	(11.96)	(16.17)	(7.27)	(25.75)	(16.26)
*n*	21,232	21,232	21,232	21,232	21,232	21,232
Sargan-Hansen χ2(3)	0.5116	0.2493	0.4828	0.2687	2.24	3.817
*First stage*						
F (4,3773)	9.59***					
F (4,3772)		11.54***				
F (4,3773)			8.11***			
F (4,3772)				12.76***		
F (4,3773)					12.23***	
F (4,3772)						14.98***

Robust cluster t statistics in parentheses

^*^p < 0.1; **p < 0.05; ***p < 0.01

^a^p < 0.111

The regression results in Table 2 indicate that for the fixed effects panel data analysis an association between poorer mental health and lower satisfaction with partners is identified for both males and females. The negative sign is consistent with lower mental health (a higher GHQ12 score) coinciding with less satisfaction with partners. The results also show consistent evidence that a higher level of an individual’s satisfaction with their partner is also associated with a higher level of their partners satisfaction with them. Similarly, an individual’s lower mental health is also associated with a lower satisfaction of their partner with them.

The instrumental variable fixed effects panel data analysis reported in Table 3 also shows that in the case when the partners satisfaction with them is excluded from the analysis, for males only, a deterioration in mental health causally reduces their satisfaction with partners. Furthermore, although when their partners satisfaction is included in the analysis this relationship becomes insignificant, the result remains close to conventional significance levels at 11.1 percent. Moreover, when their partners mental health is included in the analysis for males there is a statistically significant causal effect of a decrease in the mental health of a male reducing satisfaction with their partner. Overall, these results suggest that for females, satisfaction with partners is more likely to be a key driver of mental health because of the general significance of the associations in the non-instrumental variable regressions, but, in contrast, there is some evidence that reduced mental health can also reduce satisfaction with partners for males.

The instrumental variable results also show that for males and females a partners’ satisfaction with them remains positively associated with their satisfaction with their partner. However, the influence of a partners’ mental health on an individual’s satisfaction with their partner also becomes insignificant. These results suggest that satisfaction with partners is the key conduit with which mental health states can become shared by couples once the causal influence of individuals’ mental health on their satisfaction with partners is identified.

The results also demonstrate a ubiquitous negative association between age and satisfaction with partners and the number of children aged greater than three years in the household. Given the mean age of the sample, the former results are consistent with the literature which suggests that middle aged couples experience a dip in their marital quality [52], while the latter results support the long established negative correlation between the presence of children and marital quality [57].

## Discussion

The above results identify a ubiquitous influence of cross-partner satisfaction. The potential for a difference between males and females in the influence of mental health on satisfaction with partners, moreover, supports previous evidence in the literature. It has been noted for example that for females marital discord adds more to stress [29]. Moreover, for males, increased symptoms of depression can mediate the impact of stress on relationship satisfaction more [30]. Such differences in results between males and females identified above could be due to differences in the sense of association between partners. It has been shown that females are more inclined to make use of emotional regulation strategies to mitigate, for example, depressive symptoms [58]. They also have access to wider social networks that can provide psychological support [59]. In this sense, relationship building and satisfaction generally, as well as with partners, is integral to mental health.

However, for males it has also been shown that relationships are affected by their perceived independence and not needing emotional support [60]. For example, it has been suggested that a male strategy for coping with depressive symptoms can often involve engaging in increased activity that takes them further from their partners both behaviourally and emotionally. This would reduce the satisfaction with their partner [60, 61].

The study adds to the literature, thus, in demonstrating that in a longitudinal context mental health is an important feature of a happy and satisfied personal relationship for dyadic partners in the UK. However, the analysis also reveals that for males, poorer mental health has the potential to exacerbate feelings of lower satisfaction with partners and hence their well-being. These results are important as they offer supporting evidence for the WHO and the recent NHS policy arguments that health can be viewed as an asset that underpins the well-being and flourishing of individuals. The results indicate, thus, that investment in mental health support in the UK could have intersectoral and cross-cutting impacts on other social care domains such as relationship counselling which need to be accounted for in planning [62, 63]. Such investment would help to create a virtuous circle within households and further improve mental health and well-being. This is because whilst the above analysis identifies some evidence of a causal impact of mental health on the satisfaction with partners for males only, nonetheless the latter is important for both male and female mental health.

This study has limitations. The analysis is based on the UK and hence the generalisation of the results needs further investigation because it is known that gender differences in mental illness vary internationally [64]. Constrained by the availability of the data, the choice of instruments was restricted to lagged value of mental health, and the dummy variables for the countries in the UK which are selected to capture elements of the national variation in supply of health. Although the shortcomings of the selected instruments have been addressed through appropriate diagnostic statistics with acceptable results, exploration of more focussed sets of instruments would help if available in other data sets. With more disaggregated data instruments could also include measures of the actual supply-side of care represented by the provision of health care services in the individuals’ locality. This would provide insight into which specific services might influence outcomes. To do this, requires the use of spatial and potentially primary data. One logistic cost, however, is likely to be a reduction in the duration of the longitudinal data.

Future research might also focus in a more nuanced way on the pathways in which mental health might influence marital quality. As noted above, the GHQ12 is an aggregate measure of mental health to which there are distinct components measuring, for example, depression, confidence, being under strain etc. [43]. Moreover, in the USS other dimensions of marital quality than satisfaction are measured such as dyadic cohesion (through the working together on a project), or affective expression (such as the frequency of kissing a partner). The literature indicates that strain could affect satisfaction [30] which can lead to depression [52]. In this regard, the relationship between items in the GHQ12 and different dimensions of marital quality should be explored.

It has also been argued, for example, that well-being is highest for married individuals, and successively declines for those that are cohabiting, dating, single, widowed and divorced [65]. Moreover, in a dyadic context relationship quality has been shown to reflect these findings such that married couples report the highest relationship quality and cohabitors without marriage plans the lowest marital quality [66]. Future research might explore with greater granularity the satisfaction with partners experienced in different forms of relationships compared to the married and civil partnerships explored in this paper. It might also focus on the transitions between the forms of relationship and the role of satisfaction with partners on the transition. For example, it is known that cohabitation increasingly precedes marriage especially for those earlier in the life course [67]. Finally, future research might also further explore the cross-partner influences and interactions of mental health and satisfaction with partners on outcomes for each partner. A structural equation modelling strategy unpicking the pathways of influence would be relevant to all of these endeavours.

## Conclusions

Current UK health policy increasingly seeks to identify health as an asset, playing a role in an integrated care system seeking to promote a more inclusive and productive society, with a focus on the general flourishing of individuals. The literature also recognises the importance of personal relationships in the promotion of health and calls for further longitudinal analysis and causal inference. This study has undertaken the task of addressing causality between mental health and the satisfaction with personal relationship based on a nationally represented longitudinal data in the UK. This is achieved by applying fixed effects instrumental variable panel data estimation.

The analysis above shows that investing in mental health as an asset can improve the well-being of individuals through raising satisfaction with partners which can be amplified across partnerships. Such investment, it is shown, is particularly important for males for whom there is some evidence that poorer mental health can reduce satisfaction with personal relationships thus potentially creating a vicious circle of decline.

## Data Availability

The datasets analysed during the current study are available from the UK Data Archive Study Number 6614 https://beta.ukdataservice.ac.uk/datacatalogue/studies/study?id=6614, https://doi.org/10.5255/UKDA-SN-6614-12.

## Abbreviations

SDH: Social Determinants of Health
BHPS: British Household Panel Survey
USS: Understanding Society Survey
GHQ12: 12 Item General Health Questionnaire
WHO: World Health Organisation
UK: United Kingdom
NHS: National Health Service
